# Marionette lines correction with volumizing threads

**DOI:** 10.1111/jocd.16490

**Published:** 2024-07-23

**Authors:** Kyu‐Ho Yi, Soo Yeon Park

**Affiliations:** ^1^ Division in Anatomy and Developmental Biology, Department of Oral Biology Human Identification Research Institute, BK21 FOUR Project, Yonsei University College of Dentistry Seoul Korea; ^2^ Maylin Clinic (Apgujeong) Seoul Korea; ^3^ Made‐Young Plastic Surgery Clinic Seoul Korea

**Keywords:** filler, jowl, marionette line, prejowl sulcus, volumizing thread

## 1 INTRODUCTION

The aging of the face is a multifaceted phenomenon characterized by substantial modifications in different facial structures. Alterations in the skin, including diminished collagen and depletion of subcutaneous fat, coupled with changes in the facial skeleton and soft tissues, collectively contribute to this evolutionary process.^1^, ^2^, ^3^, ^4^ Currently, there is a diverse range of facial rejuvenation techniques accessible to counteract the effects of aging. These popular procedures, encompassing fillers, toxins, thread lifting, lipofilling, and laser treatments, are in high demand.^5^, ^6^

Within these minimally invasive interventions, thread lifting emerges as a popular modality for facial rejuvenation. Originally conceived as a facelift technique, thread lifting is now widely recognized as a creative and practical approach to skin rejuvenation by practitioners.^7^, ^8^, ^9^, ^10^

Current research underscores the dependability of thread lifting as a minimally invasive approach, highlighting prompt recovery, elevated patient contentment, and minimal risk of adverse effects.^9^, ^10^, ^11^, ^12^, ^13^, ^14^

The aging process is marked by a noticeable reduction in facial volume, which significantly influences the definition of the jawline. This phenomenon results in skin sagging, the formation of jowls, and the emergence of prominent marionette lines. Given the pivotal role of the jawline in facial attractiveness, especially in adults, a holistic approach to facial rejuvenation is imperative. This approach involves a thorough assessment and targeted interventions to address concerns related to the jawline.

In cosmetic procedures, a common goal is to address the aging effects, particularly evident in facial features, like marionette lines. Also referred to as melomental folds, these lines are a consequence of facial aging, presenting as downward‐curving wrinkles originating from the corners of the mouth. Traditionally, marionette lines were associated with repetitive facial movements and the impact of gravity on less elastic skin tissue. The cosmetic treatment of well‐established marionette lines has historically posed challenges. However, there is a noticeable shift away from employing more invasive volumizing thread methods to address these lines, with a preference for less invasive procedures that demonstrate increased efficacy. The utilization of threads has exhibited promise in improving the appearance of marionette lines.

**FIGURE 1:**
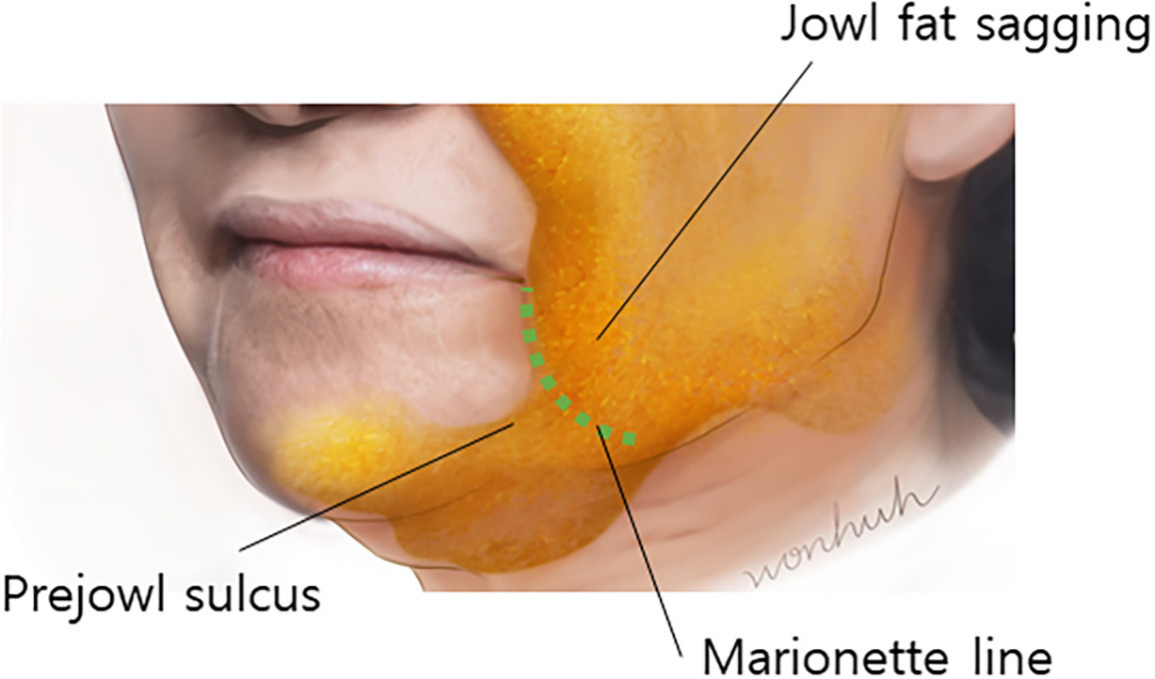
The emergence of the marionette line becomes noticeable with the elongation of the nasolabial fold. This is commonly marked by a decrease in subcutaneous fat beneath the inner lower part of the fold and the gathering of fat above the outer upper part. This leads to a clear delineation, aided by the mandibular ligament at the lowest point of the marionette line. The ligament tightens the tissues within the fold, limiting their mobility and emphasizing the boundary.

The marionette line becomes apparent as the lengthening of the nasolabial fold occurs, typically characterized by a reduction in subcutaneous fat beneath the inner lower aspect of the fold and the accumulation of fat (jowl fat) above the outer upper aspect. This results in a distinct demarcation, facilitated by the mandibular ligament at the lowermost point of the marionette line, which tightens the tissues within the fold, restricting their movement and accentuating the boundary (Figure 1). In such cases, correction of volume asymmetry is often addressed through procedures such as liposuction, transplantation, or filler injections. Specifically, introducing volumizing agents into the vertical direction of the marionette line can mitigate the visibility of the boundary between the two fat layers.

## 2 CROSS TECHNIQUE WITH VOLUMIZING THREAD

**FIGURE 2:**
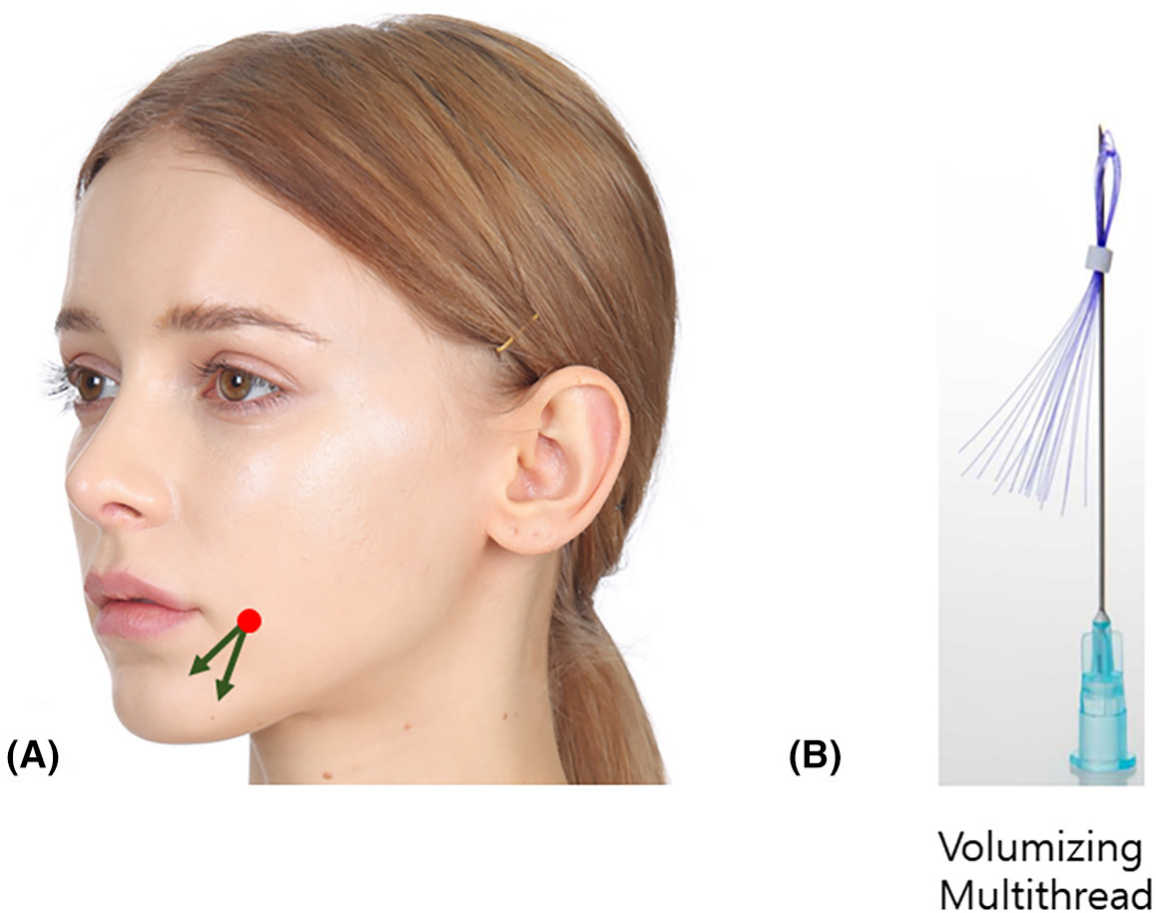
The vector for inserting volumizing threads (LVDR Volume, Sihler Inc., South Korea and Volume S Secret, Hyundaimeditech Inc., South Korea).

The technique involves placing volumizing threads perpendicular to the marionette line, strategically positioned to minimize the visibility of the boundary between the two layers of fat (Figure 2). While conventional multi‐threads, composed of numerous monothreads, experience diminished volumizing efficacy due to compression by surrounding tissues upon insertion. Volumizing threads, characterized by multiple monothreads that contribute to reduced pain during procedures and mitigate damage to surrounding blood vessels and tissues, consequently minimizing bruising, swelling, and pain.

For the cross technique utilizing volumizing threads, entry points are established inside or outside the marionette line, with specific locations determined by the length of the volumizing threads being used. Inserting the threads across the marionette line along the entry points result in the volumizing thread section within the thick tissue beneath the marionette line providing support, while the upper portion of the thread above the marionette line compresses and pushes the loose skin tissue, creating a lifting effect. This method of inserting volumizing threads vertically along the marionette line promotes collagen growth along the thread direction, tightening the loose skin tissues above the marionette line. Consequently, it reduces the degree to which the skin around the mouth is pressed and folded by facial muscles during expressions. Caution is advised against inserting the threads too deeply, as it may potentially lead to protrusion of the threads into the oral cavity.

To employ the cross technique using volumizing threads, an entry point is established inside the marionette line, and its specific location is determined based on the length of the volumizing threads to be used. Inserting the volumizing threads across the marionette line, from bottom to top, through the entry point, the section of the volumizing thread beneath the marionette line enters the thick tissue, providing a supportive base. Simultaneously, the upper part of the volumizing thread exerts pressure on and pushes the loose skin tissue above the marionette line. Placing volumizing threads vertically along the marionette line prompts collagen growth along the thread direction, firming the loose skin tissues above the marionette line. Consequently, when facial muscles move the area around the mouth, the skin around the corner of the mouth is pressed and folded to a lesser extent.

When inserting volumizing threads inside the marionette line, care must be taken to avoid injuring the mental artery and nerve emanating from the mental foramen below the corner of the mouth. The mental foramen often lies approximately midway along a vertical line drawn from the corner of the mouth to the mandible's border.

**FIGURE 3:**
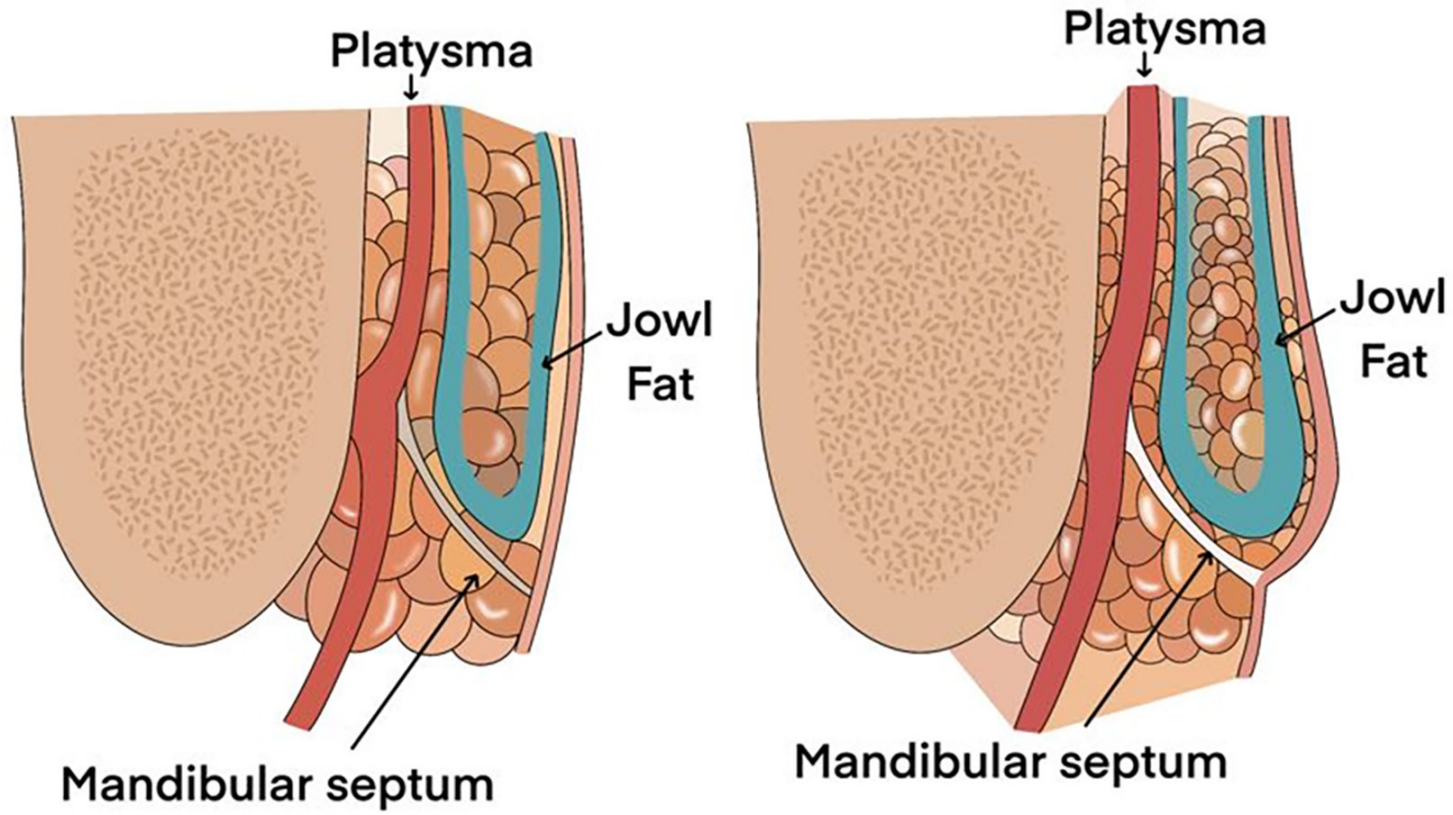
Typically, fat beneath the inner lower portion of the wrinkle line diminishes while fat (jowl fat) accumulates above the outer upper part, creating a defined boundary. Additionally, the mandibular ligament at the lowest part of the marionette lines pulls the tissues within the wrinkle line inward, preventing them from shifting and further accentuating the boundary.

In elderly patients with sagging lower facial skin and associated tissues, the prejowl sulcus may accompany the marionette line, causing the area beneath the wrinkle line to appear compressed, resulting in an uneven jawline. The outer region beyond the jaw's tip has a thin subcutaneous fat layer compared to the jaw's tip, and the mandibular ligament along the bone's jawline attaches to the skin, providing a firm pull (Figure 3). Due to this fibrous tissue, if the prejowl sulcus appears significantly compressed, achieving smooth restoration with fillers alone can be challenging. In such cases, combining volumizing threads and fillers can yield more effective results. During the procedure, pre‐tunneling between firm fibrous tissues is recommended to create space for the adequate insertion of volumizing threads and fillers, facilitating a smooth advancement of the cannula for their insertion.

The cannula entry point is located approximately at the border between the chin and the mandible body, around the visibly depressed area's anterior boundary. Needle puncture is performed at this site, followed by lateral insertion of the cannula, guiding the volumizing thread along the depressor anguli oris and platysma muscles, or the subcutaneous fat layer. After inserting the volumizing thread, any uneven areas are addressed by injecting filler into the dermal or subcutaneous tissue through the same puncture site, ensuring a smoother appearance.

By lifting the sagging tissues outside the marionette line and revitalizing the depressed area in front of the wrinkle line, the overall contour around the corner of the mouth improves. For achieving a softer transition between firm and lax tissue boundaries and reducing the folded lines at the border of these tissues, a more supple volumizing thread or monofilament can be employed. When inserting threads parallel to the wrinkle line, placing them slightly inside the wrinkle line can mitigate differences in the skin layer caused by the depressed area. Placing volumizing threads or monofilaments along the wrinkle line encourages collagen growth along the thread direction, firming the skin tissues and reducing symptoms of skin compression and folding in the area in front of the wrinkle line.

### 2.1 Cogged thread lifting

In elderly patients, sagging of the lower facial skin and connective tissues contributes not only to the marionette line itself but also to the concurrent presence of the prejowl sulcus. This results in the area below the wrinkle line appearing compressed, giving an uneven appearance to the jawline, with the region above the wrinkle line appearing more pronounced. The outer region beyond the jawline tends to have thinner subcutaneous fat layers compared to the chin tip, and the mandibular ligament, running along the bone's jawline, attaches firmly to the skin. This ligament pulls and tightens the skin, creating a firm grip.

**FIGURE 4:**
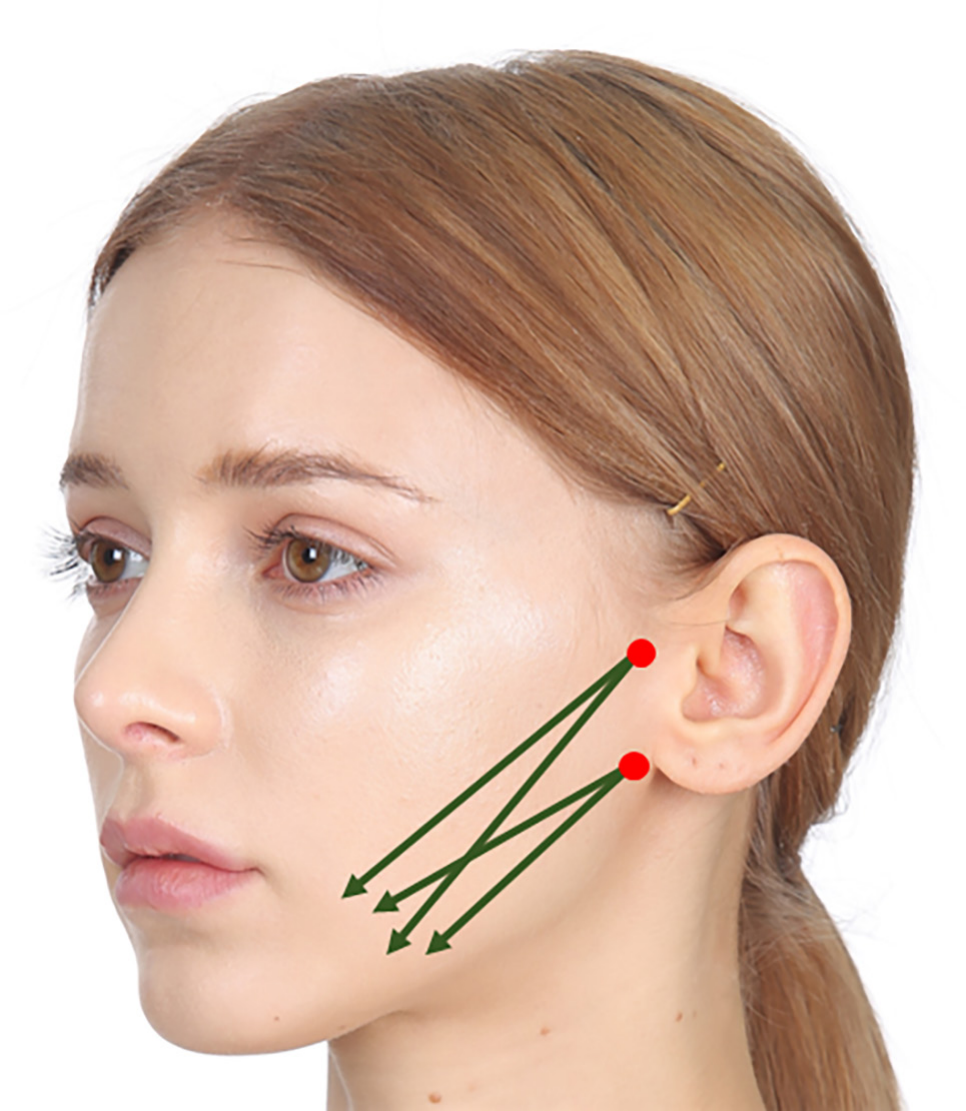
To rectify the displaced fat that contributes to jowling and attain a more balanced outcome, it is recommended to combine oblique cog thread lifting with vertical lifting along the wrinkle line, guiding the threads towards the ear region. (LVDR Lift, Sihler Inc., Korea and Secret Illusion, Hyundaimeditech Inc., Korea).

To address the displaced fat contributing to jowling and achieve a more harmonious result, it is advisable to perform oblique cog thread lifting in conjunction with vertical lifting along the wrinkle line, directing the threads towards the ear region (Figure 4). This simultaneous approach helps target the specific fat deposits responsible for jowling, providing a comprehensive solution for a more aesthetically pleasing outcome.

### 2.2 Volumizing prejowl sulcus

**FIGURE 5:**
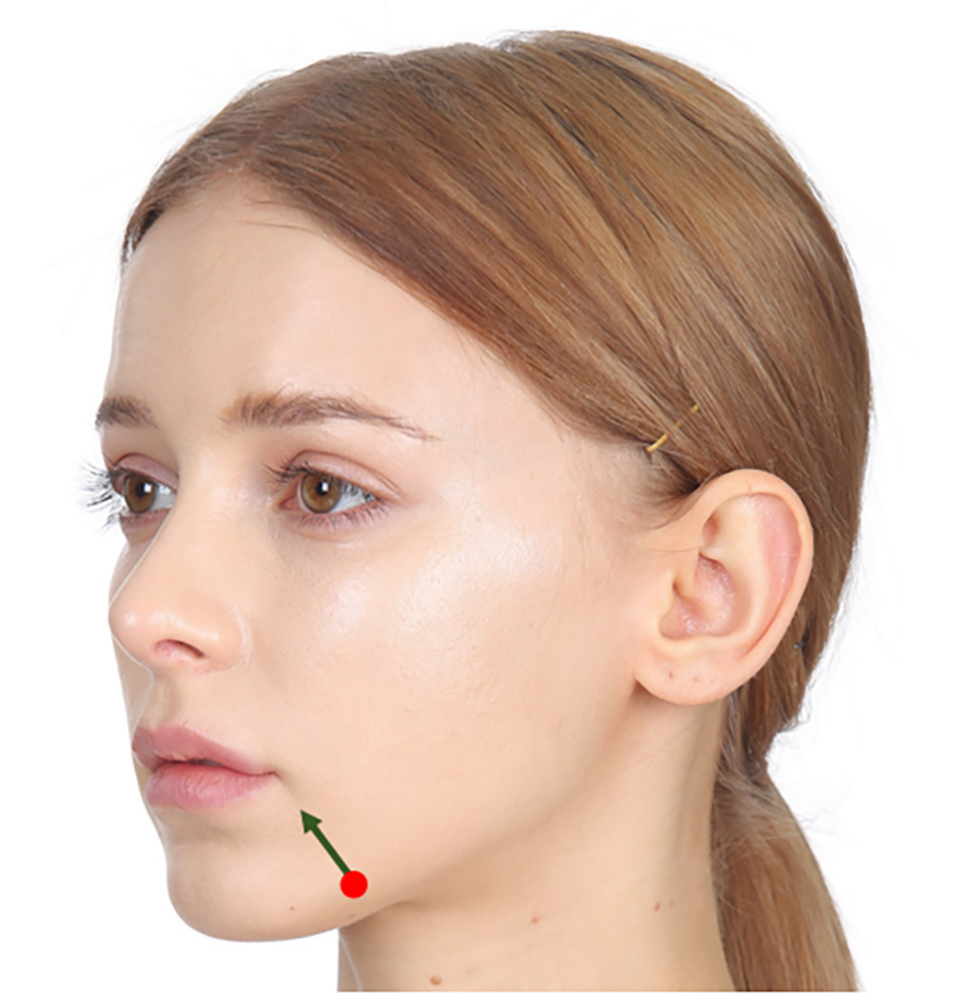
In instances where the prejowl sulcus exhibits notable compression attributed to ligamentous tissues, obtaining a more favorable result can be enhanced by simultaneously utilizing volumizing threads and fillers in the affected region. (LVDR Volume, Sihler Inc., South Korea and Volume S Secret, Hyundaimeditech Inc., South Korea).

In cases where the prejowl sulcus itself appears significantly compressed due to ligamentous tissues, achieving a more favorable outcome can be facilitated by concurrently employing volumizing threads and fillers in the affected area (Figure 5). During the procedure, it is essential to pre‐tunnel the firm ligamentous tissues, creating adequate space for the insertion of volumizing threads and fillers. The cannula entry point is typically situated around the border area between the chin tip and mandible body, where the region appears compressed and receded. Following needle puncture at the front boundary of the compressed area, the cannula is inserted outwardly to place volumizing threads along the depressor anguli oris and platysma m. or the submuscular fat layer. Irregularities post volumizing thread insertion can be addressed by injecting fillers into the dermal or subcutaneous tissue through the same puncture site, promoting a smoother appearance.

**FIGURE 6:**
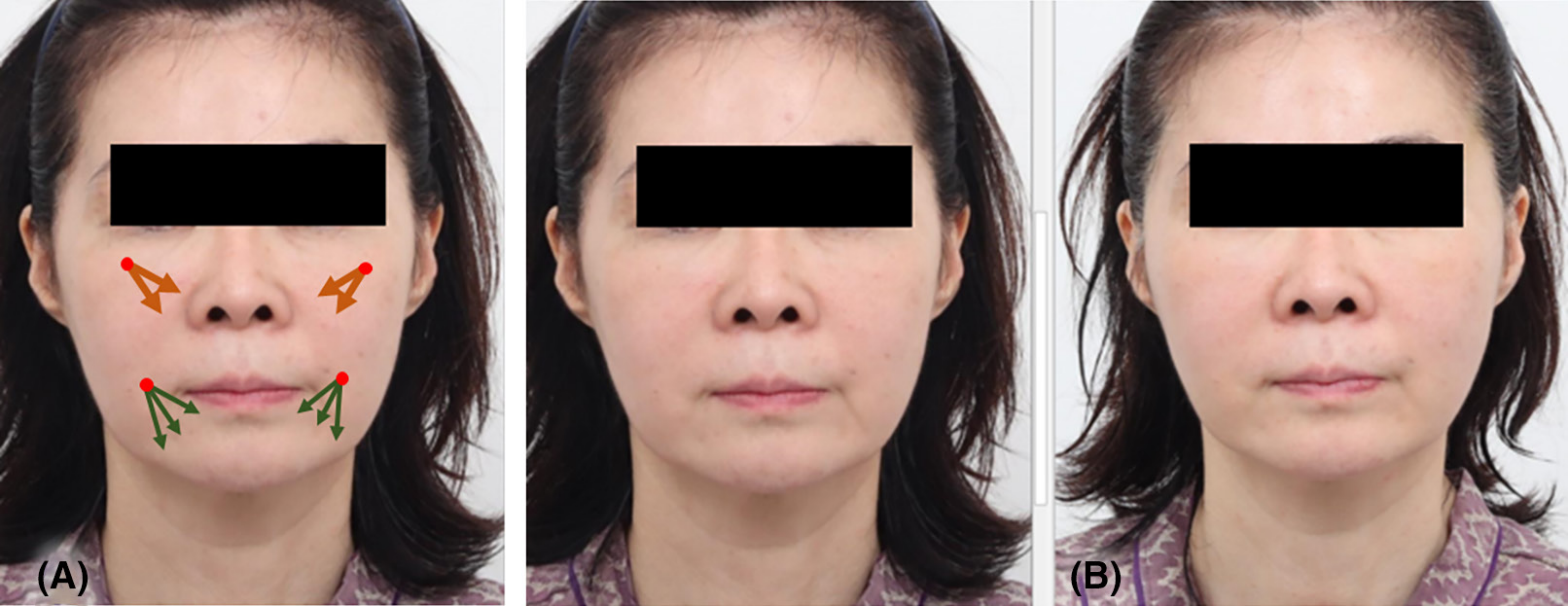
Fundamentally, it is essential to acknowledge that utilizing various threads beyond basic single vectors and threads enables a more enduring thread lifting effect. Specifically, the introduction of volumizing threads in a cross‐pattern that traverse both tissues diminishes the gap between sagging tissue with jowling on one side, promoting adhesion and considerably prolonging the duration. (LVDR Volume, Sihler Inc., South Korea and Volume S Secret, Hyundaimeditech Inc., South Korea).

In essence, it is crucial to recognize that employing diverse threads beyond simple single vectors and threads allows for longer lasting thread lifting (Figure 6). Particularly, the insertion of cross‐pattern volumizing threads that traverse both tissues reduces the disparity between sagging tissue with jowling on one side, inducing adhesion and significantly extending the duration. Additionally, for refining the overall contour after repositioning sagging tissues beyond the marionette line and revitalizing the depressed areas in front of the wrinkle line, a softer form of volumizing threads or monofilaments may be utilized. When inserting threads parallel to the wrinkle line, placing them slightly inward compared to the wrinkle line helps improve the difference in skin layers due to the compressed areas. The insertion of volumizing threads or monofilaments along the wrinkle line encourages collagen growth in the thread direction, tightening the skin tissues and reducing symptoms of skin folding in front of the wrinkle line. The utilization of various threads aims to induce synergy among the inserted threads, enhancing overall effectiveness.

## 3 CASE 1

**FIGURE 7:**
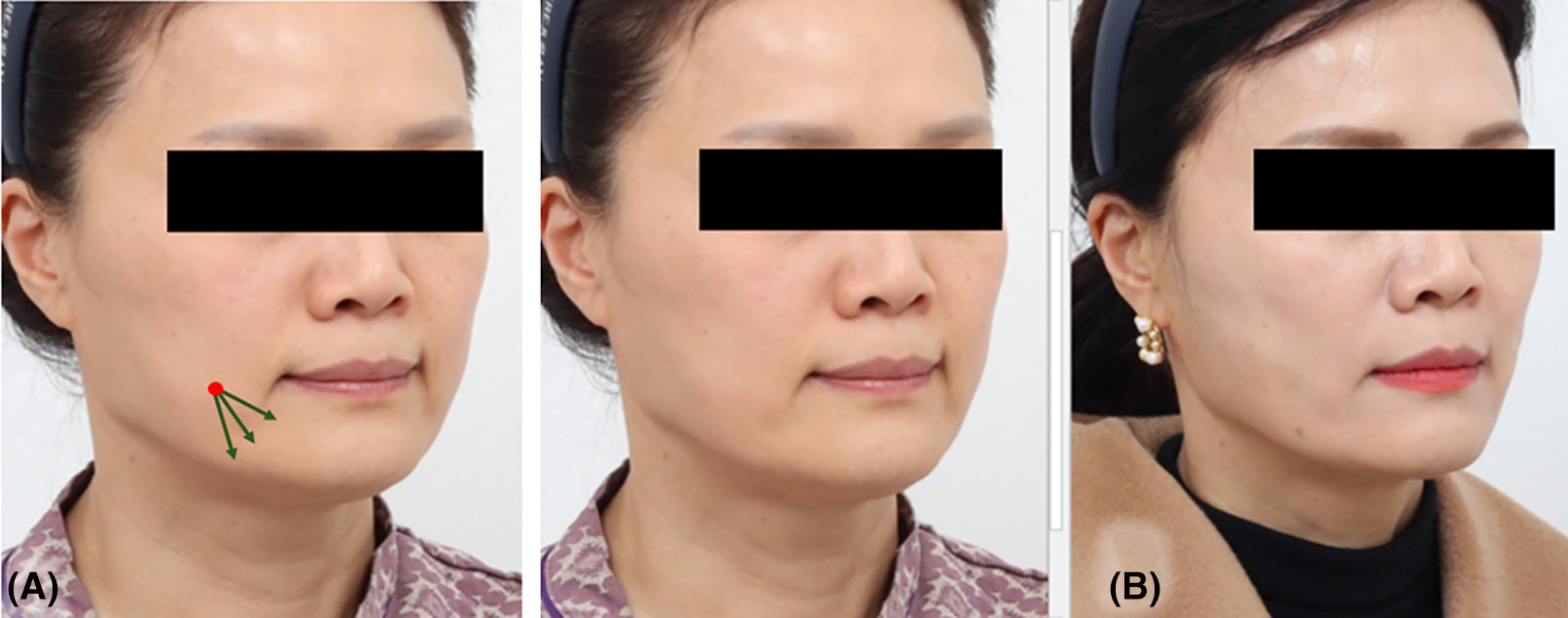
A 53‐year‐old woman expressed distress about her marionette line. The procedure entailed vertically inserting two rows of 27G volumizing threads along the marionette line wrinkle. This targeted placement effectively promoted collagen production along the thread orientation, resulting in the firming of skin tissues and improvement in symptoms related to skin compression and folding. The threads were meticulously placed into the subcutaneous layer using an 18G needle after an initial puncture. Point of entry (marked in red) and 27G volumizing thread (indicated by the dark green arrow). Prior to the treatment (A) and 4 months post‐treatment (B). (LVDR Volume, Sihler Inc., South Korea and Volume S Secret, Hyundaimeditech Inc., South Korea).

A 53‐year‐old female presented with a concern related to the marionette line. Comparative images were captured in both the pre‐procedural and 4‐month post‐procedural stages (Figure 7). The intervention involved the vertical insertion of two lines of 27G volumizng polydioxanone threads (LVDR Volume, Sihler Inc., South Korea and Volume S Secret, Hyundaimeditech Inc., South Korea) along the marionette line wrinkle. This placement strategically stimulated collagen production along the thread direction, leading to the tightening of skin tissues and the amelioration of symptoms associated with skin compression and folding. 0.1 cc of dental lidocaine was injected at the entry site prior to the insertion of the thread. The threads were precisely introduced into the subcutaneous layer, facilitated by an 18G needle following an initial puncture. The thread was inserted into the subcutaneous layer (layer II) while pressing the jowl flat with the palm. Cannula was retracted with rotating the cannula at the end of the insertion.

## 4 CASE 2

**FIGURE 8:**
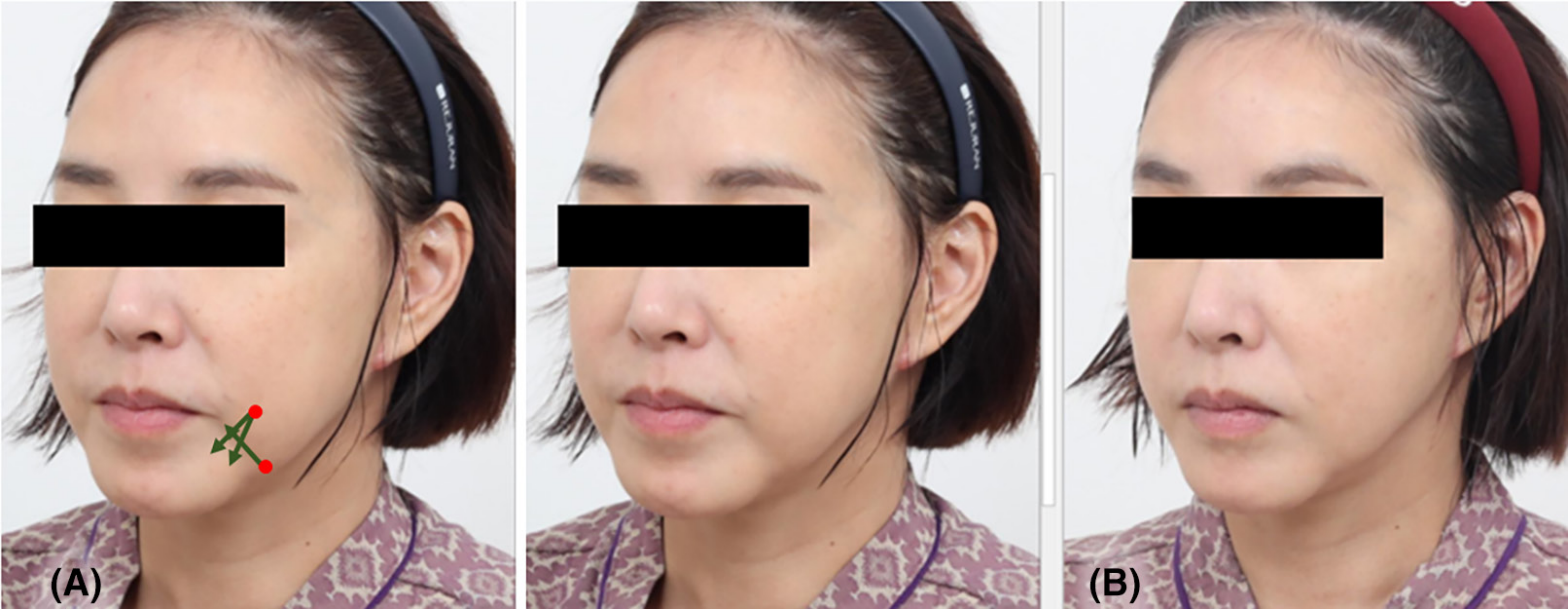
A 55‐year‐old female voiced concerns about marionette lines. Utilizing 21G volumizing threads in the anterior cheek region and introducing two pairs of 27G volumizing threads vertically along the marionette line wrinkle resulted in noticeable improvements in both the volumizing effects of the anterior cheek and the refinement of the marionette line. Point of entry (marked in red), 21G volumizing thread (indicated by the dark orange arrow), and 27G volumizing thread (indicated by the dark green arrow). Prior to the treatment (A) and 2 months post‐treatment (B).

A 55‐year‐old woman expressed a marionette line‐related concern. Comparative photographs were taken before the procedure and 2 months after the procedure (Figure 8). In the anterior cheek area, 21G volumizng polydioxanone threads (LVDR Volume, Sihler, Korea) were introduced, and along the marionette line wrinkle, two sets of 27G volumizng polydioxanone threads (LVDR Volume, Sihler Inc., South Korea and Volume S Secret, Hyundaimeditech Inc., South Korea) were vertically inserted. Before inserting the thread, 0.1 cc of dental lidocaine was administered at the entry site. A puncture was then created with an 18G needle and the thread was placed at the subcutaneous level (layer II). The cannula was retracted by rotating it at the end of the insertion. Evident enhancements were observed in both the volumizing effects of the anterior cheek and the refinement of the marionette line.

## 5 CASE 3

A 46‐year‐old female patient, with a history of facelift surgery several years ago, sought thread lifting for correction of the marionette line and prejowl sulcus. For this purpose, 27G volumizng polydioxanone threads (LVDR Volume, Sihler Inc., South Korea and Volume S Secret, Hyundaimeditech Inc., South Korea) were inserted vertically along the marionette line in two lines, and one line each was inserted directly into the prejowl sulcus. Prior to threading, 0.1 cc of dental lidocaine was injected at the entry point. An 18 G needle was used to create a puncture and the thread was then inserted into the subcutaneous layer (layer II). The cannula was retracted by rotating it at the end of the insertion.

Improvement in both areas was observed. Post‐procedure photos were taken 6 months later.

## 6 DISCUSSION

Typically, the reduction of fat occurs on the inner lower side of the wrinkle line, while fat (jowl fat) accumulates on the outer upper side, creating a distinct boundary. Additionally, the presence of the mandibular ligament at the lowest point of the marionette line causes the tissues on the inner side of the wrinkle line to contract, preventing them from moving and resulting in a clear demarcation.^15^

In such cases, correction is achieved through procedures like liposuction, grafting, or filler injections to address the asymmetry in volume. This involves elevating the cheek area as much as possible, reducing volume, creating elasticity, and augmenting volume on the medial side.

Contemporary aesthetic clinics have witnessed a surge in the popularity of knotless thread lifting devices utilizing absorbable threads. Despite its widespread adoption, a notable drawback of this procedure lies in its applicability to only a moderate degree of facial tissue laxity.^16^, ^17^, ^18^

The development of marionette lines, influenced by facial anatomy and aging dynamics, manifests as visible sagging around the mouth. These lines, influenced by factors like muscle composition and tissue depth variations, contribute to a downturned appearance reminiscent of a marionette puppet's mouth. Effectively addressing these lines often involves a comprehensive approach, employing minimally invasive procedures like thread lifting and fillers to mitigate sagging and enhance facial aesthetics. Techniques emphasizing specific anatomical considerations and precise thread insertion have demonstrated promise in effectively treating and minimizing these lines, thereby reducing risks and improving outcomes.

Favorable anatomical characteristics for absorbable thread lifting include a low body mass index, minimal soft tissue fullness, robust underlying bony structures providing support, and good skin quality. Conversely, individuals with obesity and thicker soft tissues may yield less favorable results, underscoring the importance of meticulous patient selection for optimal outcomes.

To combat significant skin sagging, the strategic insertion of 1–3 additional cog threads along the cheek has proven effective. Concurrently, addressing additional marionette lines and lifting the chin involves using 3–5 extra cog threads perpendicular to the chin line, resulting in more favorable outcomes.

The thread technique boasts advantages such as avoiding general anesthesia and scarring due to its incision‐free nature. It has demonstrated efficacy in treating uneven facial textures, midface slackness, and mild to moderate jowls in specific patient groups. Complications associated with this procedure are infrequent and generally minor, establishing the utilization of threads for aesthetic facial rejuvenation and lifting as a safe method.

## AUTHOR CONTRIBUTIONS

All authors have reviewed and approved the article for submission. *Conceptualization*: Kyu‐Ho Yi and Soo Yeon Park. *Writing—original draft preparation*: Kyu‐Ho Yi and Soo Yeon Park. *Writing—review and editing*: Kyu‐Ho Yi and Soo Yeon Park. *Visualization*: Kyu‐Ho Yi and Soo‐Yeon Park. *Supervision*: Hong, Kyu‐Ho Yi and Soo‐Yeon Park.

## FUNDING INFORMATION

There is no financial disclosure to report.

## CONFLICT OF INTEREST STATEMENT

I acknowledge that I have considered the conflict of interest statement included in the “Author Guidelines.” I hereby certify that, to the best of my knowledge, that no aspect of my current personal or professional situation might reasonably be expected to significantly affect my views on the subject I am presenting.

## ETHICS STATEMENT

This study was performed in line with the principles of the Declaration of Helsinki.

## Data Availability

The data that support the findings of this study are available from the corresponding author upon reasonable request.
